# Incidence and Spread of *Mycobacterium tuberculosis*-associated Infection among Aba Federal Prison Inmates in Nigeria

**DOI:** 10.3329/jhpn.v28i4.6038

**Published:** 2010-08

**Authors:** Lawrence N. Chigbu, Christian U. Iroegbu

**Affiliations:** ^1^ Department of Microbiology, College of Medicine, Abia State University, Aba, Abia state, Nigeria; ^2^ Department of Microbiology, University of Nigeria, Nsukka, Enugu state, Nigeria

**Keywords:** HIV, *Mycobacterium tuberculosis*, Prisoners, Tuberculosis, Tuberculin skin test, Nigeria

## Abstract

The study was undertaken to determine transmission of *Mycobacterium tuberculosis* within the prison environment. In total, 168 Aba Federal prison inmates in Nigeria were evaluated for tuberculosis (TB) by sputum-smear microscopy and sputum culture, simultaneously, and for HIV status by serology. They were subsequently followed up for one year for fresh *Mycobacterium*-associated infection by tuberculin skin testing or for development of TB and for HIV infection or AIDS. Ninety-one (54.2%) of the 168 prison inmates had infection due to *Mycobacterium*, and three (3.3%) of them were sputum-smear- and culture-positive while 41 (24.4%), including one (2.4%) with concomitant TB, were HIV-infected. In a one-year follow-up study, 11 (19.3%) of 57 tuberculin skin test (TST)- and HIV-negative inmates became TST-positive and one (1.8%) HIV-positive, eight (13.8%) of the 58 TST-positive but HIV-negative inmates developed TB, and one (1.7%) became HIV-infected: six (24.0%) of 25 TST- and HIV-positive inmates developed TB while five (33.3%) of 15 TST-negative but HIV-positive inmates became TST-positive, and one (6.7%) progressed to AIDS. The duration of imprisonment did not influence the rates of infection, and the transmission of *Mycobacterium tuberculosis* did not necessarily require sharing a cell with a TB case.

## INTRODUCTION

Tuberculosis (TB), one of the most important contagious diseases, is a leading cause of death due to a single pathogen worldwide. Patients with pulmonary TB would broadcast the tubercle bacilli in droplet aerosols as they cough, sneeze, or even talk and infect those in contact. A person with untreated pulmonary TB is estimated, on average, to infect 10-15 persons annually (1). A primary infection due to *Mycobacterium tuberculosis* may actively develop into clinical TB, pass as inapparent infection, or remain latent in the individual for months or years depending on the various host and environmental factors. Overt TB, thus, could result from a reactivated latent infection or from a recent primary infection or (secondary) re-infection. It has been observed that the transmission of *M. tuberculosis* is favoured by dusty environment and overcrowding. Infections can be acquired by both close and casual contacts. The risk of becoming infected depends on such factors as the relative virulence of the strain, the intensity of exposure to an infectious TB case (closeness and duration), and the susceptibility and immune status of the exposed individual (2,3). Mitsos *et al*. observed that only a small proportion of individuals infected with *M. tuberculosis* develop clinical TB (4), and even then, a wide clinical spectrum of severity of disease is observed in such individuals. A lifestyle, such as tobacco/cigarette smoking, could increase the chances of developing clinical TB four-fold (5–7) due to the various effects of smoking on components of both innate and adaptive immunity (8–11). Exposure to indoor air pollution has been associated with TB among other broncho-pulmonary diseases (12). Epidemiologic studies have shown that risk of TB increases with close contacts of sputum-smear-positive patients and that the prevalence of clinical disease among intimate contacts of TB cases is high (13–15).

HIV infection has become an additional factor that has specifically threatened TB programmes worldwide, and for the last two decades, the HIV pandemic and TB have been inextricably linked. TB has become the most common cause of death among HIV-infected adults in less-developed countries (16). In sub-Saharan Africa where ≈70% of HIV-infected individuals of the world live, the annual rate of identification of TB cases has quadrupled since the mid-1980s (17), and over two-thirds of TB patients in the region are dually infected with HIV (18). It is estimated that about 200,000 deaths due to TB occur among the people of sub-Saharan Africa concomitantly infected with HIV (19).

The factors influencing infection and progression to TB and even AIDS tend to prevail in crowded institutions, such as military camps, prisons, and police detention camps. The Nigerian prisons seem most favourable for the dissemination of *M. tuberculosis* and progression to TB and transmission of HIV and progression to AIDS given the overcrowding in cells, poor feeding, and allegations of homosexual practices and sexual abuse among the incarcerated. It was on account of these that the investigation reported here was carried out among the inmates of the Federal prisons in Aba, Nigeria.

## MATERIALS AND METHODS

### Sample

The sample consisted of 168 male prisoners, aged 20-63 years (mean age 33.8 years), who had been incarcerated from two days to 13 years. They were accommodated in four cells designated C, D, H, and I respectively, each measuring about 12.2 m × 12.2 m × 3.7 m. There were 65 inmates in Cell C, 35 in Cell D, 26 in Cell H, and 42 in Cell I. The prisoners were allowed two hours each to come out and stretch their limbs, bask themselves in sunshine, and breathe fresh air or more hours if they were assigned to outdoor labour. Feeding was generally poor, and food from relatives and Church organizations or charities visiting the prisons was permitted following a strict screening for safety. Individual characteristics of the prisoners, such as lifestyle, history of smoking, alcohol consumption, sexual behaviours, and history of TB or contact with TB patient, were sought and voluntarily given by the inmates. A Prisons Medical Unit with trained paramedical personnel in charge treated minor ailments and referred cases with persistent respiratory symptoms to the hospital. The investigation started with inmates brought to the Chest Clinic of the ABSUTH for clinical evaluation and treatment from June to November 2006. They were followed to the prison, and other inmates were investigated either for similar symptoms or as contacts.

### Collection and examination of sputum

Early-morning sputum specimens were obtained from the patients in three consecutive days in sterile wide-mouthed disposable containers (Sterilin). The sputum was processed using a modification of the Baltimore Biological Laboratories (BBL) method (20). To the sputum in a test-tube was added an equal volume of 4% (w/v) NaOH containing phenol red indicator, vortexed to homogenize, and stored at room temperature (≈27°C) for 30 minutes to digest. The mixture was subsequently neutralized with an equal volume of 2N HCl and centrifuged at 3,000 rpm for five minutes in a Gallenkamp bench-top centrifuge. The supernatant was discarded, and a portion of the sediment was used for making three smears on three clean grease-free slides, air-dried, and heat-fixed. The smears were then stained with concentrated carbol-fuschin (primary stain), steamed for five minutes in the first instance, and further for three minutes after adding fresh stain. Thereafter, the slides were rinsed with water, decolourized with 3% HCL/ethanol mixture until the stain was no more visible, and then counter-stained with methylene blue for two minutes before rinsing with water and drying for microscopic examination.

### Isolation and characterization of *Mycobacterium tuberculosis* from sputum specimens

Both NaOH-digested and undigested sputum specimens were inoculated onto separate screw-capped bottles of the modified Lowenstein-Jensen (L-J) egg medium prepared as follows: first, a mineral salt solution, containing KH_2_PO_4_ (0.4%^w^/_v_), MgSO_4_.7H_2_O (0.4%^w^/_v_), magnesium citrate (0.1%^w^/_v_), asparagines (0.6%^w^/_v_), and glycerol (2%^v^/_v_), was prepared with distilled water and sterilized by autoclaving at 121°C for 15 minutes. To 300 mL of this solution was added 10-20 mL of sterile 2% Malachite green solution and the contents of 12-16 freshly-laid hen's eggs (aseptically). The mixture was homogenized in a previously-sterilized Warren blender, and the homogenate was dispensed in 6-7 mL volumes into sterile screw-capped disposable universal bottles (Sterilin). The L-J medium so dispensed was further sterilized by inspissations for 45 minutes in slanted position in a water bath at 85°C.

Each inoculated L-J medium was incubated at 37°C for 8-10 weeks, observing it daily for growth. Cultures showing no significant growth after 10 weeks were discarded. Smears made from the emergent colonies on the L-J slopes were stained by the Ziehl-Neelsen method and examined microscopically for acid-fast bacilli (AFB). AFB-positive colonies were further tested for nitrate reduction, niacin and catalase production respectively and for hydrolysis of Tween-80 at the TB Unit of the Nigerian Institute for Medical Research, Yaba, Lagos, using standard bacteriological techniques.

### Screening for exposure to *Mycobacteriun* species by tuberculin skin test

The tuberculin skin test (TST) was done using the Mantoux method. Approximately 0.1 mL of 5 tuberculin units per litre (5 TU/L) of PPD (Evans Media, USA) was inoculated intradermally on top of the fore arm ≈6 cm below the elbow. The site of inoculation was examined for the development of indurations 72 hours after as recommended by the American Thoracic Society and the Centers for Disease Control and Prevention (21). For individuals who tested HIV-negative, skin indurations of ≥10 mm in diameter were recorded as TST-positive (TST^+^) while those with indurations of <10 mm in diameter were recorded as negative. For the HIV-positive individuals, indurations of ≥5 mm in diameter were taken as TST^+^ but those of <5 mm were recorded TST^-^.

### Screening of patients for HIV antibodies

Approximately 4.0 mL of venous blood was collected and allowed to clot in a sterile disposable tube. The resulting serum was aspirated, centrifuged to clarify, and assayed for presence of HIV antibodies using Immunocomb II (Organics Ltd., Yavne 70650, Israel) and ELISA (SUDS HIV 1 and 2; Murex Diagnostic) test-kits.

### Follow-up study for TST/TB and HIV/AIDS

A one-year (December 2006–January 2007) follow-up study was carried out with the prison inmates. Those diagnosed with concomitant TB and HIV infection were monitored for progression to AIDS, and those who were TST-positive but HIV-negative were followed to see the development of TB and/or HIV antibodies. Those who were HIV-positive but TST-negative were followed to see the progression to AIDS and/or TST-positive reaction or TB. Finally, those who were concomitantly positive for TST and HIV were monitored for progression to TB or AIDS or both.

### Ethical clearance

The study was carried out with permission of the Nigerian Prisons Service Corps, the Abia State University Teaching Hospital Research Board, and the University of Nigeria Biosafety Committee, after inspection of the facilities at the Infectious Diseases Hospital, Aba, Abia state, Nigeria, where the study was done.

## RESULTS

Of the 168 prison inmates investigated, three (1.8%) were TB cases—one each in Cell C, H, and I, and none in Cell D. The TB case in Cell H had concomitant TB and HIV infection. Of the 65 inmates in Cell C sharing the accommodation with the single TB case, 38 (58.5%) reacted positive to TST, and seven (10.8%) of these were also HIV-positive. Cell H contained 24 inmates in addition to the TB case, and of them, 10 (41.7%) were TST-positive, and three (11.5%) of these TST-positive individuals were HIV-infected. In Cell D housing 35 inmates, among whom was no TB case, 20 (57.1%) tested TST-positive, with 13 (37.1%) of them being concomitantly infected with HIV and seven (20.0%) singly HIV-positive. In Cell I with one TB case and 41 other inmates (contacts), 20 (47.6%) of the latter reacted TST-positive, and two (4.8%) of them were concomitantly infected with HIV (Table 1).

**Table 1.** Distribution of TB/tuberculin skin test-positive and HIV-infected prison inmates by prison cells

Prison cell	No. screened	No. (%) of inmates positive					Total	
		TB^+^	TB^+^ with HIV^+^	TST^+^	TST^+^HIV^+^	HIV^+^	TB^+^/TST^+^*	HIV^+^$
C	65	1 (1.5)	0 (0.0)	31 (47.7)	7 (10.8)	6 (9.2)	39 (60.0)	13 (20.0)
D	35	0 (0.0)	0 (0.0)	7 (20.0)	13 (37.1)	7 (20.0)	20 (57.1)	20 (57.1)
H	26	0 (0.0)	1 (3.8)	7 (26.9)	3 (11.5)	2 (7.7)	11 (42.3)	6 (23.1)
I	42	1 (2.4)	0 (0.0)	18 (42.9)	2 (4.8)	0 (0.0)	21 (50.0)	2 (4.8)
Total	168	2 (1.2)	1 (0.6)	63 (37.5)	25 (14.9)	15 (8.9)	91 (54.2)	41 (24.4)

*All TB- and TST-positive cases with or without HIV infection;

$All HIV-positive cases with or without TB or M. *tuberculosis-*associated infection;

HIV=Human immunodeficiency virus;

TB=Tuberculosis;

TST=Tuberculin skin test

Table 2 shows the age distribution of the TB cases, concomitantly TB- and HIV-positive, TST-positive, concomitantly TST- and HIV-positive, singly TST- and HIV-positive individuals. Of the three diagnosed TB cases, one (2.7%) was among the 37 inmates in the age-group of 18-26 years, and two (3.8%), including one concomitantly infected by HIV, came from the group of 80 inmates aged 27-35 years. The highest frequency (66.7%) of the TST-positive individuals, including four (19.0%) who tested HIV-positive, came from the group aged over 44 years.

**Table 2.** Age distribution of TB/tuberculin skin test and or HIV-positive cases among prison inmates

Age range (years)	No. screened	No. (%) of inmates positive					Total	
		TB^+^	TB^+^HIV^+^	TST^+^	TST^+^HIV^+^	HIV^+^	TB^+^/TST^+^*	HIV^+^$
18-26	37	1 (2.7)	0 (0.0)	17 (45.9)	3 (8.1)	3 (8.1)	21 (56.8)	6 (16.2)
27-35	80	1 (1.3)	1 (1.3)	24 (30.0)	14 (17.5)	5 (6.3)	40 (50.0)	19 (23.8)
36-44	30	0 (0.0)	0 (0.0)	12 (40.0)	4 (13.3)	5 (16.7)	16 (53.3)	9 (30.0)
>44	21	0 (0.0)	0 (0.0)	10 (47.6)	4 (19.0)	2 (9.5)	14 (66.7)	6 (28.6)
Total	168	2 (1.2)	1 (0.6)	63 (37.5)	25 (14.9)	15 (8.9)	91 (54.2)	40 (23.8)

*All TB- and TST-positive cases with or without HIV infection;

$All HIV-positive cases with or without TB or M. *tuberculosis-*associated infection;

HIV=Human immunodeficiency virus;

TB=Tuberculosis;

TST=Tuberculin skin test

Table 3 shows the distribution of the TB/TST and HIV-positive inmates according to the duration of stay (months) in the prison at the time of the study. The lowest proportion (39.1%) of the inmates who tested TST-positive was among those who had stayed for ≤6 months, and the highest (75.0%) was among 12 inmates who had stayed for 42-48 months. The 23 inmates who had stayed for ≤6 months (but not shown in Table 3) included 11 (47.8%) inmates who had spent between two days and three months in the prison. Of the latter, three (27.3%) tested TST^+^, including the inmate who spent only two days in the prison, three (27.3%) were singly HIV^+^, and one (9.1%) was concomitantly TST^+^ with HIV^+^. Of the three diagnosed TB-positive individuals, one in Cell C had spent seven months in the prison, one nine months in Cell H, and the third three years in Cell I. The range of duration of stay in the prison was two days to 13 years, and the mean duration was 2.7 years.

**Table 3.** Distribution of TB/tuberculin skin test-positive and HIV-infected prison inmates by duration of stay in prison

Duration (months) of stay	No. screened	No. (%) of inmates positive					Total	
		TB^+^	TB^+^HIV^+^	TST^+^	TST^+^HIV^+^	HIV^+^	TB^+^/TST^+^*	HIV^+^$
≤6	23	0 (0.0)	0 (0.0)	8 (34.8)	1 (4.3)	4 (17.4)	9 (39.1)	5 (21.7)
7-12	29	1 (3.4)	1 (3.4)	6 (20.7)	3 (10.3)	1 (3.4)	11 (37.9)	5 (17.2)
13-18	12	0 (0.0)	0 (0.0)	7 (50.3)	1 (8.3)	0 (0.0)	8 (66.7)	1 (8.3)
19-24	20	0 (0.0)	0 (0.0)	11(55.0)	1 (5.0)	1 (5.0)	12 (60.0)	2 (10.0)
25-30	5	0 (0.0)	0 (0.0)	1 (20.0)	1 (20.0)	1 (20.0)	2 (40.0)	2 (40.0)
31-36	37	1 (2.7)	0 (0.0)	8 (21.6)	11 (29.7)	3 (8.1)	20 (54.1)	14 (37.8)
37-42	7	0 (0.0)	0 (0.0)	0 (0.0)	3 (42.9)	1 (14.3)	3 (42.9)	4 (57.1)
42-48	12	0 (0.0)	0 (0.0)	6 (50.0)	3 (25.0)	0 (0.0)	9 (75.0)	3 (25.0)
>48	23	0 (0.0)	0 (0.0)	11(47.8)	1 (4.3)	0 (0.0)	12 (52.2)	1 (4.3)
Total	168	2 (1.2)	1 (0.6)	58(34.5)	25 (14.9)	11 (6.5)	86 (51.2)	37 (22.0)

*All TB- and TST-positive cases with or without HIV infection;

$All HIV-positive cases with or without TB or M. *tuberculosis*-associated infection;

HIV=Human immunodeficiency virus;

TB=Tuberculosis;

TST=Tuberculin skin test

All the 168 inmates were also enlisted for follow-up but 155 (91.7%) were followed till January 2007, 12 (7.1%) dropped on account of their discharge from the prison, and one (0.6%)—the TB-positive inmate in Cell I with concomitant HIV infection—died. Of the 155 inmates who completed the study, 83 (53.5%) were those who were TST-positive, including those who were concomitantly positive for TST and HIV, 15 were singly HIV-positive, and 57 were negative for both TST and HIV. Of the 83 TST-positive inmates, 14 (16.9%) developed TB confirmed by sputum-smear microscopy. These included six (24.0%) of the 25 inmates who initially reacted positive to both TST and HIV. Thus, eight (13.2%) were from a group of 58 inmates who tested TST-positive but were HIV-negative at the beginning of the follow-up study. Of the 15 inmates who were singly HIV-positive at the beginning of follow-up, five (33.3%) became TST-positive, one (6.7%) developed AIDS, and none developed clinical TB. Of the 57 inmates who reacted negative to both TST and HIV, 11 (19.3%) became TST-positive and one (1.8%) HIV-positive (Table 4).

**Table 4.** Distribution of TB/tuberculin skin test-positive and HIV-infected individuals after a one-year follow-up of prison inmates with varying tuberculin skin test and HIV status

Initial disease/ infection status	No. examined	No. (%) of inmates testing positive			
		TST^+^	TB^+^	HIV^+^	AIDS
TST^-^ with HIV^-^	57	11 (19.3)	0 (0.0)	1 (1.8)	0 (0.0)
TST^+^ with HIV^-^	58	NA	8 (13.8)	1 (1.7)	0 (0.0)
TST^+^ with HIV^+^	25	NA	6 (24.0)	NA	0 (0.0)
TST^-^ with HIV^+^	15	5 (33.3)	0 (0.0)	NA	1 (6.7)
Total	155	16 (10.3)	14 (9.0)	2 (1.3)	1 (0.6)

AIDS=Acquired immunodeficiency syndrome;

HIV=Human immunodeficiency virus;

NA=Not applicable;

TB=Tuberculosis;

TST=Tuberculin skin test

## DISCUSSION

There were three diagnosed cases of TB among the 168 prison inmates investigated, and contacts of these inmates with 165 other prisoners would presumably put the latter at risk of infection with *M. tuberculosis*. It is not certain what proportion of the 52.4% other prisoners who tested TST-positive did so due to exposure to these three TB cases but 10.3% of the TST-negative inmates in the one-year follow-up study, who became positive, strongly indicate transmission of *M. tuberculosis*-associated infection from TB cases to non-TB inmates within the prison. The TST-positive case rate (47.6%) in Cell D, which had no TB case, is within the range observed in cells with TB cases (Cell C–58.5%; Cell H–38.4%, and Cell I–47.7%). Thus, transmission did not require sleeping in the same room with a TB case. It would occur at other levels of contact and intimacy, including sharing work, eating, and recreational environment. Some inmates were already exposed to infection due to *M. tuberculosis* outside before imprisonment given that four (36.4%) of the 11 inmates who had spent only ≤3 months in the prison at the time of screening, including the new inmate who had spent only two days, were TST-positive.

In addition to the transmission occurring in the prison as indicated by the earlier-mentioned 10.3% TST-negative inmates who became TST-positive within the one-year follow-up period, 33.3% of the TST^-^ with HIV^+^ inmates who became TST-positive (i.e. TST^+^ with HIV^+^) compared to 19.3% from the TST^-^ with HIV^-^ individuals presumably reflect the impact of HIV on transmission of *M. tuberculosis* among contacts of the TB cases. HIV infection was also observed to influence progression of TST-positive state to overt TB in the 24.0% TST^+^ with HIV^+^ inmates who developed TB in the one-year follow-up period compared to 13.2% of the TST^+^ with HIV^-^ individuals. In 1993, it was estimated that about 9% of global TB cases were attributable to HIV infection, and it was forecast that this would rise to 14% by 2000 (22).

Although the inmates were not screened for HIV at the inception of their incarceration, two of the 115 HIV-negative individuals enlisted in the one-year follow-up study became HIV-positive, and these are noteworthy because one had spent one year and another had spent two years at the time of initial screening when they tested negative. Thus, the transmission of HIV was also apparent among the inmates, which may be either through secret drug-use or through homosexual intercourse. This further suggests that the high rate (24.3%) of HIV observed among the Aba Federal Prison inmates, may not all be infections acquired while outside the prison but some may have been acquired while in the prison. Although lifestyles, such as tobacco/cigarette smoking and alcohol consumption, could increase the chances of developing clinical TB four-fold (5–7), with the limited data in this study, similar conclusions could not be drawn. Considering the duration of stay in the prison and the incidence rate of TB or *M. tuberculosis* and/or HIV infections, there seemed to be no significant difference between groups of different durations of imprisonment (χ^2^_cal_=2.24, p<0.05), suggesting that *M. tuberculosis* and HIV infections individually may be single-hit events that may not be dependent on the duration of contacts by inmates.

## ACKNOWLEDGEMENTS

The assistance rendered by Dr. C. Onubuogu of the National Institute for Medical Research, Yaba, Lagos, Assistant Controller of the Federal Prisons, Aba; Mrs. Ihechi Akoma and other staff of the Health Unit of the Federal Prisons, Aba; and Mrs. Iwuoha of the Infectious Diseases Hospital is gratefully acknowledged.
